# Multilevel Evaluation of Rapid Weight Loss in Wrestling and Taekwondo

**DOI:** 10.3389/fsoc.2021.637671

**Published:** 2021-04-09

**Authors:** Cecilia Castor-Praga, Jeanette M. Lopez-Walle, Javier Sanchez-Lopez

**Affiliations:** ^1^Facultad de Organización Deportiva, Universidad Autónoma de Nuevo León, San Nicolás de los Garza, Mexico; ^2^Centro de Investigación en Ciencias Cognitivas, Universidad Autónoma del Estado de Morelos, Cuernavaca, Mexico

**Keywords:** acute weight loss, wrestling, taekwondo, multidimensional evaluation, psycho-educational intervention

## Abstract

The practice of strategies for rapid weight loss (RWL) involve diverse factors, such as individual expectations, social interactions, structural elements, etc., conforming to a “culture” of RWL, which must be evaluated and understood in a broad sense. Based on the need of a comprehensive evaluation of the use of RWL in practitioners of combat sports, an *ad hoc* questionnaire designed for this study, which includes the types and detailed descriptions of RWL strategies, that athletes currently use, the prevalence and frequency of use, the physiological and psychological consequences, the perception of the effect of RWL on their own performance and finally, the individuals who influence the adoption of this practice. One hundred and sixty combat athletes from wrestling and taekwondo disciplines, from Mexico, filled out this questionnaire. Data collected for their statistical analyses. Results revealed a RWL strategies prevalence of 96% across the participants. Our results revealed that 57% of those athletes using RWL lose more than 5% of their body mass. Across the athletes, the most commonly used RWL strategies and with higher intensity were increased exercise and training with plastic or thick clothes. The greater the relative weight loss, the greater the presence of physiological symptoms in athletes, such as rapid breathing and blood pressure. Athletes also mentioned mood states such as tiredness, sadness, confusion, fatigue and vigor, these last two positive and negative mood states are associated with the relative weight loss, respectively. Finally, the people who most influenced the adoption of RWL strategies were the coaches, parents and nutritionists. In conclusion, the questionnaire prepared for this study allowed us to obtain valuable information about the several factors, and their interactions, involved in the practice of RWL in combat athletes. This type of practice could increase health risks and decrease their performance. Therefore, here we state the importance of a comprehensive evaluation of RWL strategies that allows the development of psycho-educational and social-based interventions and programs for the promotion of proper weight maintenance, and prevention against RWL strategies, involving the individuals who influence the adoption of these practices and supporting it with the help of communication technologies.

## Introduction

Combat sports are internationally practiced, and they are characterized by categorizing athletes by body mass (BM) into weight divisions or classes to minimize differences in size and strength among competitors. To ensure that athletes fulfill the weight requirements, an official weigh-in is done before the competition. The weigh-in procedures are different between the diverse Olympic combat sports, such as: judo, taekwondo (tkd), boxing, and wrestling (Reale et al., 2018). The purpose of weight classes is to match athletes with similar body build to create an equal level of competition and minimize the risk of injury between opponents (Jetton et al., 2013).

It is known that combat athletes perform strategies for Rapid Weight Loss (RWL) which is an acute loss of BM in the week prior to the competition. The magnitude of the percentage of weight athlete's loss varies depending on the time they implement the strategy before the official weigh-in. RWL occurs often (Artioli et al., 2010b; Brito et al., 2012; Franchini et al., 2012; Khodaee et al., 2015) with the aim to qualify in a class lower than the athletes training weight, seeking to gain an advantage by competing against lighter, smaller and weaker opponents (Artioli et al., 2010d; Fernández-Elías et al., 2014; Reale et al., 2016b) These practices take place within a structural, social and cultural context. There are studies that tell us about the adoption of the practices, how the rules are accepted within the sports community becoming a key part of the sport and, as a consequence, these practices are well-established.

However, there is the belief that RWL is related to mental advantages during the competition. This has been evidenced by reports from athletes performing RWL that indicate a sense of “sporting identity” or “real sportsman” as consequence of achieving weight reduction quickly (Hall and Lane, 2001; Koral and Dosseville, 2008; Jetton et al., 2013; Pettersson et al., 2013; Reale et al., 2016a). In this regards, one study has reported that competitive success is positively associated with RWL (Reale et al., 2016a), however, most studies have reported that the success is mainly due to years of experience of the athlete (Hall and Lane, 2001; Artioli et al., 2010c; Kazemi et al., 2011; Brito et al., 2012; Franchini et al., 2012; Zubac et al., 2017; Reale et al., 2018) and not the amount of weight he/she has lost and the recovery, prior to weigh-in and prior to competition, respectively. The truth is that the weight control strategies often employed are at the expense of health and sport performance (Kowatari et al., 2001; Alderman et al., 2004; Degoutte et al., 2006; Prouteau et al., 2006; Green et al., 2007; Artioli et al., 2010b; Pettersson et al., 2013; Reljic et al., 2016; Nascimento-Carvalho et al., 2018). There is variability in the prevalence, methods and magnitude of weight loss, as well as recovery post RWL, among sport disciplines.

Concerns about acute health risks from the continued use of RWL have mainly focused on the loss of more than 5% of BM by means of extreme dehydration or food deprivation on days 1 or 2 prior to weigh-in. It has been suggested that a reduction of 5% or less in BM does not significantly affect performance, as long as the athlete has a few hours to feed and rehydrate after weigh-in (Artioli et al., 2010b,c; Franchini et al., 2012). Despite this, the prevalence of those individuals losing more than 5% of weight and the number of days prior to which they begin to use strategies for RWL have not been reported. And the athletes who lose and gain weight intentionally and constantly (weight cycling), as a result of the practice of RWL (Saarni et al., 2006) has a negative effect on physical performance. This may be due to poor replenishment of the overall intake of macronutrients by athletes on competitions day as they do not replenish their overall needs of macronutrients and water between official weigh in and the competitions (Artioli et al., 2010a; Pettersson and Berg, 2014).

The risk produced by RWL depends on a combination of factors, such as the amount of reduced BM, time for this reduction, and the frequency of episodes and/or strategies used for RWL (Artioli et al., 2010b). To achieve RWL athletes use a combination of several potentially harmful methods, such as severe restriction of intake of food and liquids, exercising with plastic or heavy clothing, use of saunas, taking diet pills, or even vomiting (Alderman et al., 2004). Although there are various strategies for RWL, dehydration and food restriction are the most common methods and, together, result in alterations in body fluid and the availability of glycogen (Oppliger et al., 2003; Kordi et al., 2011; Franchini et al., 2012; Reale et al., 2016b).

Despite the well-documented adverse effects of RWL on health status, the prevalence of aggressive and harmful procedures for rapidly weight reduction is very high in most combat sports such as wrestling (Steen et al., 1988; Kiningham and Gorenflo, 2001; Oppliger et al., 2003, 2006; Alderman et al., 2004) and tkd (Kazemi et al., 2011; Brito et al., 2012). Extreme dehydration can cause a decrease in plasma volume, resulting in a decrease in systolic volume, an increase in heart rate, and a decrease in the difference in arteriovenous oxygen during submaximal exercise (Rankin, 2002), which harm performance and can also be hazardous to health (Jetton et al., 2013).

In addition, dehydration has adverse effects such as alteration of the central nervous system, increases in central temperature, cardiovascular stress due to glycogen deficiency and alterations in metabolic function (Cheuvront et al., 2003; Artioli et al., 2010b). Side effects have also been reported from the use of short-term RWL strategies (that is, they affect the health of the athlete during the weight reduction period) presenting negative emotions (Degoutte et al., 2006) and sensations such as muscle fatigue, and/or symptoms of weakness, muscle pain and/or myalgia and depression (Kordi et al., 2011; Zubac et al., 2017). A long-term effect is that athletes experience higher rates of obesity later in life (Saarni et al., 2006), problems of anxiety, perfectionism and eating disorders (more studied in women), and irregular menstruation in women (Filaire et al., 2007; Rouveix et al., 2007; Escobar-Molina et al., 2015) due to reduced baseline metabolic rate, making weight maintenance difficult (Nascimento-Carvalho et al., 2018).

Other consequences are depressed autoimmune activity making athletes more susceptible to disease, reduced bone density and injuries (Kowatari et al., 2001; Prouteau et al., 2006; Green et al., 2007; Rouveix et al., 2007).

Alterations in the growth of athletes and hormonal imbalance (Reljic et al., 2016), as well as cognitive functions have also been reported (Nascimento-Carvalho et al., 2018). Considering the negative physical, emotional, and psychological effects of RWL, presumably it affects sports performance negatively, however, there are few studies measuring the effect of RWL on sport performance.

Excessive weight reduction practices during adolescence could affect body development. For instance, these practices have been reported in individuals between nine and 17 years old, although RWL has also been observed in athletes younger than seven years old; that is the reported case of a 5 year old athlete encouraged by his father (Oppliger et al., 2003; Sansone and Sawyer, 2005; Kordi et al., 2011; Brito et al., 2012).

People with the greatest influence in teaching and adopting strategies for RWL are regularly the coaches, sport mates, former athletes; while the least influential tend to be the parents, physicians and dietitians (Oppliger et al., 2003; Reale et al., 2018). Some authors propose that the culture of the sport is also a major influence for quick weight reduction and that the athletes are resistant to change this practice (Kordi et al., 2011; Reale et al., 2018). Structural factors in sport rules influencing the use of RWL are the weight rating system, the programming patterns and the organization of events (Artioli et al., 2010a; Zubac et al., 2017) as well as the physiological requirements of sport (Lagan-Evans et al., 2011). It is known that RWL strategies are less used by athletes of higher weight categories, meaning that the lower the category the higher the prevalence and aggressiveness of use of strategies for RWL (Kiningham and Gorenflo, 2001; Alderman et al., 2004; Artioli et al., 2010b; Reale et al., 2016b).

Finally, no previous studies have reported the use and type of strategies utilized by athletes, the symptoms and their perception in relation to sport performance, the characteristics of the context and the actors that facilitate the RWL in a comprehensive way that allows us to understand it as a complex phenomenon in order to generate appropriate actions to prevent and contain it and its negative consequences.

Therefore, the aim of this study was to implement a holistic instrument to evaluate contextual (agents and performance perception) and psychophysiological (physiological and psychological symptoms) variables with respect to the prevalence and methods of RWL, correlated with the amount of weight loss and the relation in wrestling and tkd athletes.

## Materials and Methods

There were 160 combat sport athletes (48 wrestling and 112 tkd), 96 men and 64 women belonging to Sport Center from Mexico. Athletes of both sexes between seven and 24 years old (13.34 ± 2.89), that is, infant and youth categories, height 156.61 ± 12.67 cm and BM 48.89 ± 15.29 kg, the weight divisions were from 39 to 125 kilograms in wrestling and 27 to 68 kilograms in tkd. Participants with at least 1 year of competitive experience and accepted informed consent were included. Evaluation was conducted during the pre-competitive period. In terms of sports age, the average was 6.55 ± 2.62 years, while for the competitive age it was 5.39 ± 2.29 years. All procedures were carried out in accordance with the ethical principles and Standards of the Declaration of Helsinki 1964 and its subsequent amendments on human research, and the current version of Ley General de Salud de Mexico. This study was approved by the Ethical Committee of Centro de Investigación Transdisciplinar en Psicología of the Universidad Autónoma del Estado de Morelos.

### Instruments

Based on the previous literature, a questionnaire was created to obtain qualitative and quantitative data for the detection of the type of strategies on RWL practices that athletes have used and use, the frequency and intensity of their use, and its consequences as a change of mood and feelings and perception of sports performance during the season when experiencing weight changes and symptoms of dehydration, as well as the social environment promoting the RWL practice.

The questionnaire consists of 4 sections. The section one was based on the questionnaire of Artioli et al. (2010d) *Rapid Weight Loss Questionnaire* (RWLQ) validated with Brazilian judo athletes with Cronbach's scores of 0.98 obtained between test and retest, which was based on other questionnaires made for wrestlers (Steen et al., 1988; Kiningham and Gorenflo, 2001; Oppliger et al., 2003; Alderman et al., 2004) and the previous research of Artioli et al. (2007) and Zubac et al. (2017). Most of the questions focus on generic information (diet history, weight management practices and competitive level). Specific questions were adapted, allowing their application to combat sports. In addition, this section collects general data as well as years of practice and competitive experience of the athletes.

Section two regarded the evaluation of the people who influence athletes to practice RWL and the intensity of their influence.

Section three consisted of the report of how the weight of athletes fluctuates from the general to the competitive period, as well as the sensations they experience when they lose weight rapidly, and the perception of how this affects their performance. This part consisted of open questions.

Finally, section four, evaluated the symptoms of dehydration (Santos-Peña et al., 2006) when performing strategies for RWL 3 to 5 days prior to the competition.

An expert review was made by coaches of wrestling, judo, boxing and karate; besides, a preliminary study with 30 wrestlers, not included in the final sample, to verify the relevance and comprehension of the questions included in the questionnaire, and the relevant adjustments were made prior to their final application (see Supplementary Material 1).

To test the factorial structure of the *Ad Hoc* Questionnaire through the present sample, in order to obtain evidence of the validity of the three scales presented above. For this purpose, we performed confirmatory factor analyses (CFAs) using AMOS program.

In order to assess the fit of these models, we examined RMSEA (root mean square error of approximation), CFI (comparative fit index), NFI (normed fit index), TLI (Tucker-Lewis's index), and AIC (Akaike information criterion) goodness of fit statistics. The interpretation of these indexes is as follows: RMSEA < 0.08 = acceptable model; CFI, NFI, and TLI > 0.90 = acceptable model, and >0.95 = excellent model; AIC values close to 0 indicate a better fit and greater parsimony (Bentler and Bonett, 1980; Bentler, 1990; Hu and Bentler, 1995; Dudgeon, 2004).

Table 1 shows the goodness of fit statistics of confirmatory factor analyses performed at the *Ad Hoc* Questionnaire.

**Table 1:** Goodness of fit statistics of confirmatory factor analyses performed at the *ad hoc* Questionnaire.

			**Absolute**			**Incremental**			**Parsimony**
**Factor**	***p***	***df***	**χ^2^/df**	**χ^2^**	**RMSEA**	**CFI**	**TLI**	**NFI**	**AIC**
Strategies	0.00	65 (104–39)	2.18	142.08	0.09	0.84	0.80	0.74	222.08
Influence of people	0.00	14 (35–21)	6.86	96.12	0.19	0.98	0.97	0.97	138.12
Symptoms	0.00	20 (44–24)	3.27	65.50	0.11	0.99	0.99	0.99	113.50

### Study Procedure

Athletes were summoned to the facilities of the Sport Center an hour before their training, they were asked to attend rested and fed. The application of the questionnaire was carried out collectively, six evaluators previously trained conducted the application, the athletes took ~40 min to answer. Prior to the application of the questionnaire, all participants were orally briefed on what the instrument was, and each participant of legal age signed the informed consent. For underage athletes the informed assent and consent for parents was granted and signed prior to the application session.

### Data Analysis

Data analysis consisted of several steps, calculation of relative weight lost, categorization of mood states related to lost weight, and the indices of success and experience were created on basis of the original items (see below). To explore the normality of the data the Shapiro-Wilks test was performed which showed that the data did not follow a normal distribution; therefore, subsequent inferential analysis was performed by means of a non-parametric statistical test. Finally, descriptive, correlational and mean comparison analyses were performed for the different variables; see below for a more detailed description.

The calculation of the new variables was performed as follows. The relative percentage of weight lost in the athletes was calculated by dividing the weight they regularly lose prior to a competition by their regular weight during the entire season. This calculation also helped us to classify the athletes as those who lose more or <5% of their BM.

Since a questionnaire collected information by mean of open questions for the sections of mood state and perception of performance, a categorization of the participant's responses were conducted *a posteriori* for statistical analysis. The qualitative items regarding the mood state related to weight lost, they were categorized in anger, confusion, sadness, fatigue, vigor and no changes from the perception of the athletes when they practiced RWL.

This procedure helps to identify the athletes that do or do not present a specific mood state. The same procedure was performed to identify whether or not those athletes perceive a decline in their sport performance. The success index was calculated by dividing the number of medals by the number of competitions they had in the last year. Finally, the sport experience index was calculated by dividing the competitive age by the sport age.

For the analyses of the strategies for RWL, two measures were considered: (a) the presence/absence of the strategy which allowed us to calculate the sample frequency for its use; and (b) the intensity in the use of the strategy categorized into rarely, sometimes and always. For the physiological symptoms, a similar procedure was performed; however, the intensity was categorized as normal, moderate and severe. Analyses were conducted considering the whole sample, but also separated into the two groups who lost more or <5% of their relative weight.

Statistical descriptive analysis consisted of the calculation of percentages, means and standard deviations for the prevalence of use of RWL strategies in the overall sample and the subgroups (more/less 5% of BM loss), subgroups by sexes and disciplines, the days prior to the competitions that they carry out RWL strategies, the amount of RWL strategies, the intensity of the type of strategies that they used, the physiological symptoms as consequence of RWL strategies, the emotional symptoms and the change in the perception of performance as consequence of RWL strategies, and the influence of others in using RWL strategies in the overall sample and in the subgroups (more/<5% of BM loss).

For the correlational analysis, we used the Spearman test, where the association between the relative weight lost with the amount and frequency of RWL strategies, the influence of people for use of RWL strategies, the success index, or the sport experience index, were tested.

Finally, comparisons were performed between groups using the U Mann-Whitney test to identify the differences in the intensity of the strategies that they use, the intensity of the symptoms as consequence of RWL and the influence of others to adopt this practice between the two subgroups (more/<5% of BM loss), both sports and sexes.

For the analysis of emotional symptoms, comparisons between groups consisted of the difference of the relative weight lost, the quantity of strategies and the intensity of the strategy between those participants reporting vs. participants not reporting the changes in mood state, therefore analyses were performed for each category of mood separately. For the comparisons between sexes and sport disciplines, the analysis was conducted by means of chi squared test for each mood state category. All the statistical analyses were performed using the software IBM SPSS v. 25.

## Results

### Prevalence of RWL

The aim of this analysis was to identify the prevalence of RWL strategies among the sample of combat athletes. We found that 96% (*n* = 153) of the participants reported use of strategies for RWL, from this sample the relative weight loss was calculated only for 148 athletes. This allows dividing the sample in two groups: 57.40% of the athletes who practice strategies for RWL lose more than 5% of their BM, while 42.60% lose <5%.

The percentage relative weight lost was 5.21 ± 3.99 and the reported days prior to competitions that they carry out RWL strategies were 9.95 ± 5.92. Finally, the range of age of onset of RWL reported was between 7 and 17 years.

A significant difference between sports were found in the greatest amount of weight they had lost along their sport career (*U* = 1,266.50, Z = −4.56, *p* < 0.001, *Hedges' g* = 0.45) where the wrestling athletes lose more than tkd athletes and the age of onset of RWL (*U* = 433.00, Z = −8.14, *p* < 0.001*, Hedges' g* = 1.59) where the wrestling athletes begin to use first the strategies for PRP than tkd athletes. No significant differences were found in terms of sex.

### Frequency and Intensity of RWL Strategies

This subsection aimed to explore the amount and intensity of RWL strategies. The frequency and the intensity of use of RWL strategies in both sports from the highest to the lowest are shown in Table 2. Despite the fact that the gradual diet, a proper and healthy form of weight reduction, is one of the most used (82.03% of which 28.12% always use it), it is observed that increased exercise and training with plastic or thick clothes and spit out, that means, dehydration strategy, are the most intense strategies (always) used for RWL. The mean amount of RWL strategies used by individuals was 5.73 ± 2.68.

**Table 2:** Percentage of athletes of wrestling and taekwondo reporting different intensity in the use of the diverse rapid weight loss strategies.

**RWL strategies**	**Athletes using strategies, currently (%)**	**Rarely (%)**	**Sometimes (%)**	**Always (%)**
Increased exercise	86.48	16.40	50.00	33.59
Training with plastic or thick clothes	75.67	14.28	55.35	30.35
Fluid restriction	74.32	38.18	40.90	20.90
Omit food	69.59	35.92	44.66	19.41
Spit out	61.48	20.87	50.54	28.57
Fasting	54.05	25.00	58.75	16.25
Intentionally training in hot rooms	54.05	38.75	45.00	16.25
Saunas	41.21	26.22	60.65	13.11
Laxatives	29.72	59.09	34.09	6.81
Use plastic or thick clothes even without training	27.70	53.65	31.70	14.63
Diuretics	10.81	62.50	37.50	0.00
Vomiting	7.80	28.57	71.42	0.00
Diet pills	3.90	50.00	50.00	0.00

The participants reporting different intensity in the use of the diverse RWL strategies are shown in Table 3. Results are separated by subgroups as function of their relative weight lost, more or <5% of BM loss. Notice that the strategies are used with higher intensity in the group with more than 5% of BM loss, except for the use of laxatives (see the last column in Table 2 “Always”).

**Table 3:** Percentage of participants reporting different intensity in the use of the diverse rapid weight loss strategies.

**RWL strategies**	**+5%**	**−5%**	**+5%**	**−5%**	**+5%**	**−5%**
	**Rarely (%)**		**Sometimes (%)**		**Always (%)**	
Increased exercise	5.65	10.48	19.35	30.65	19.35	14.52
Training with plastic or thick clothes	4.63	10.19	22.22	32.41	19.44	11.11
Spit out	5.62	14.61	22.47	28.09	19.10	10.11
Fluid restriction	8.49	29.25	18.877	21.70	16.98	4.72
Omit food	16.83	18.81	15.84	28.71	13.86	5.94
Use plastic or thick clothes even without training	17.07	36.59	14.63	17.07	12.20	2.44
Intentionally training in hot rooms	14.47	25.00	21.05	22.37	11.84	5.26
Fasting	9.09	15.58	25.97	32.47	11.69	5.19
Saunas	15.79	12.28	33.33	26.32	10.53	1.75
Laxatives	21.43	35.71	16.67	19.05	2.38	4.76
Diuretics	40.00	26.67	13.33	20.00	0.00	0.00
Vomiting	0.00	33.33	33.33	33.33	0.00	0.00
Diet pills	16.67	33.33	33.33	16.67	0.00	0.00

*RWL, Rapid Weight Loss; n = 148; results are separated by subgroups as function of their relative weight lost, more or <5% of BM loss*.

Although from the correlation analysis there was not a significant association between the relative weight lost and the amount of strategies used, a positive correlation was found between the intensity of strategies they use and relative weight lost, specifically with the use of plastics even without training (*r*_*s*_ = 0.38, *p* < 0.05) and fluid restriction (*r*_*s*_ = 0.40, *p* < 0.01), i.e., it seems that the athletes lose more weight when these strategies are used with greater intensity.

To disentangle differences in the intensity of use of RWL strategies between athletes losing more or <5% of their BM, median comparisons between groups were performed for all the strategies separately. Results are shown in Table 4 where the group with more than 5% of body mass loss displayed higher intensity in the use of five of 11 strategies relative to the group with <5% of BM loss.

**Table 4:** Comparison between subgroups as a function of their relative weight lost, more or <5% of body mass loss and the intensity of the type of rapid weight loss strategies used.

**RWL strategies**	**Athletes who lost ± 5% of body mass**					
	**More than 5%, *mean rank***	**Less than 5%, *mean rank***	***U Mann-Whitney***	***Z***	***p***	***Hedges' g***
Increased exercise	68.97	57.34	1541.50	−1.96	**0.05**	0.29
Training with plastic or thick clothes	61.57	48.41	1096.50	−2.42	**0.01**	0.01
Fluid restriction	66.69	42.99	766.50	−4.22	**0.00**	0.44
Omit food	54.03	48.36	1126.50	−1.04	0.29	
Spit out	51.62	39.09	709.00	−2.49	**0.01**	0.05
Fasting	43.03	35.46	593.00	−1.67	0.09	
Intentionally training in hot rooms	43.13	34.34	553.50	−1.87	0.06	
Saunas	30.50	26.78	340.00	−0.94	0.34	
Laxatives	22.21	21.02	200.50	−0.35	0.73	
Use plastic or thick clothes even without training	24.83	18.00	138.00	−2.01	**0.04**	1.09
Diuretics	7.38	8.71	23.00	−0.70	0.48	
Vomiting	4.50	3.00	2.00	−1.11	0.26	
Diet pills	4.00	3.00	3.00	−0.74	0.46	

*Bold values indicate significant p < 0.05*.

Differences were found between males and females where the latter have higher intensity in the use of fasting (*U* = 1,534.00, Z = −3.17, *p* = 0.002, *Hedges' g* = 0.96), laxatives (*U* = 494.50, Z = −2.48, *p* = 0.013, *Hedges' g* = 0.74) and omit food (*U* = 868.50, Z = −2.99, *p* = 0.003, *Hedges' g* = 1.19). No significant differences were found between disciplines.

### Physiological Symptoms as Consequence of RWL Strategies

In terms of physiological symptoms reported when they use RWL strategies, the frequency and the intensity for the general physiological state (i.e., sleepy, sweaty, sometimes comatose/excessive inactivity, cyanotic limbs/limbs change blue) was reported with 23.80 and 48.30% for normal, and 17.50 and 9.10% for moderate in the group of more than 5% of BM and <5% of BM, respectively, and 0.70% for severe in both groups.

The rest of the intensity of the physiological symptoms are displayed in Table 5 from highest to lowest according to the severity. As we can see, the symptoms that were presented with greater severity for the whole sample were the mucous membranes (i.e., dry or very dry mucous membranes).

**Table 5:** Percentage of athletes of wrestling and taekwondo reporting different intensity of physiological symptoms as consequences of rapid weight loss strategies.

**Symptoms**	**Normal (%)**	**Moderate (%)**	**Severe (%)**
Mucous membranes	62.30	24.70	13.00
Blood pressure	76.40	14.20	9.50
Urine	45.60	47.00	7.40
Eyes	61.30	33.30	5.30
Heart rate	70.90	27.70	1.40
Tears	75.50	23.30	1.30
Breathing	78.00	20.00	2.00

Table 6 shows the percentage of participants reporting different levels of intensity of physiological symptoms as consequence of RWL strategies between the subgroups losing more or <5% of BM, the absence of tears and lack of urine being the most severe physiological symptoms in the group that loses more than 5% of their BM. Notice that this latter group tends to show more severity in the symptoms than the group with <5% of BM loss.

**Table 6:** Percentage of athletes reporting different intensity of physiological symptoms as consequence of rapid weight loss strategies divided in subgroups of more or <5% of body mass loss.

**Symptoms**	**+5%**	**−5%**	**+5%**	**−5%**	**+5%**	**−5%**
	**Normal (%)**		**Moderate (%)**		**Severity (%)**	
Tears	62.71	60.98	18.64	29.27	18.64	9.76
Urine	70.00	81.93	15.00	12.05	15.00	6.02
Breathing	52.46	67.86	36.07	30.95	11.48	1.19
Blood pressure	63.93	76.19	26.23	20.24	9.84	3.57
Mucous membranes	45.16	43.90	45.16	50.00	9.68	6.10
Heart rate	67.74	86.75	29.03	12.05	3.23	1.20
Eyes	67.74	80.95	32.26	16.67	0.00	2.38

*The relative weight lost was not obtained from all participants, because five of them did not report the amount of weight they usually lost or the regular weight during the season (n = 148)*.

Correlational analysis revealed a significant positive association between the physiological symptoms, general physiological state (*r*_*s*_ = 0.24, *p* < 0.01), breathing (*r*_*s*_ = 0.21, *p* < 0.05) and blood pressure (*r*_*s*_ = 0.22, *p* < 0.01) with the relative weight the athletes lost. In addition, the amount of strategies used was also positively correlated with heart rate (*r*_*s*_ = 0.18, *p* < 0.05), breathing (*r*_*s*_ = −0.28, *p* < 0.01), eyes (*r*_*s*_ = 0.28, *p* < 0.01), mucous membranes (*r*_*s*_ = 0.30, *p* < 0.01), urine (*r*_*s*_ = 0.26, *p* < 0.01), and blood pressure (*r*_*s*_ = 0.30, *p* < 0.01).

Comparisons between groups were performed to test the difference in the reported intensity of the physiological symptoms between the groups with more or <5% of BM loss. Results are shown in Table 7. More severe symptoms were reported by the group with more than 5% of BM loss in the general physiological state (i.e., sleepy, sweaty, sometimes comatose/excessive inactivity, cyanotic limbs/limbs change blue), heart rate (i.e., fast and impalpable sometimes) and breathing (i.e., deep and fast).

**Table 7:** Comparison between subgroups (More vs. Less 5% of Body Mass Loss) of the intensity of physiological symptoms as consequence of RWL strategies.

**Symptoms**	**Athletes who lost ± 5% of body mass**					
	**More than 5%, *mean rank***	**Less than 5%, *mean rank***	***U Mann-Whitney***	***Z***	***p***	***Hedges' g***
General physiological state	82.88	64.13	1837.00	−3.43	**0.001**	0.59
Heart rate	80.90	67.10	2083.00	−2.75	**0.006**	0.47
Breathing	80.78	67.35	2087.00	−2.22	**0.03**	0.48
Eyes	78.73	69.64	2280.00	−1.71	0.09	
Tears	71.78	70.44	2373.00	−0.22	0.82	
Mucous membranes	72.84	72.24	2521.00	−0.09	0.92	
Urine	77.33	68.15	2170.50	−1.78	0.08	
Blood pressure	78.59	68.94	2221.00	−1.72	0.08	

*RWL, Rapid Weight Loss. Bold values indicate significant p < 0.05*.

In addition, a significant difference was found in the symptoms associated to urine (*U* = 1805.00, Z = −3.01, *p* = 0.003, *Hedges' g* = 0.35) where athletes of wrestling showed higher intensity in the symptoms than athletes of tkd. The analysis between males and females revealed differences between the symptoms associated to tears (*U* = 1929.0, Z = −3.01, *p* = 0.003, *Hedges' g* = 0.50) where men present a higher intensity than women do.

### Mood State Symptoms as Consequence of RWL Strategies

The frequency of athletes reporting the presence or absence of the diverse mood states because of the use of RWL strategies are shown in Table 8. The most frequent mood states were fatigue (i.e., tired, muscle pain, exhausted, without energy, weak), vigor (i.e., with energy, content, motivated), and confusion (i.e., disoriented).

**Table 8:** Percentage of participants reporting the presence/absence of mood states as consequence of rapid weight loss strategies.

**Mood states**	**Presence (%)**	**Absence (%)**
Anger	8.10	91.90
Confusion	17.00	83.00
Depression	2.20	97.80
Fatigue	48.90	51.10
Vigor	20.00	80.00
No changes	11.90	88.10

Table 9 shows the percentage of athletes who lost more or <5% of their body mass reporting the presence or absence of mood changes related to RWL. Notice that those athletes losing more than 5% tend to report more frequently the presence of negative mood states.

**Table 9:** Percentage of participants reporting the presence/absence of mood states as a consequence of rapid weight loss strategies divided in subgroups considering more or <5% of body mass loss.

**Mood states**	**Athletes who lost ± 5% of body mass**			
	**Less than 5% of BM loss**		**More than 5% of BM loss**	
	**Presence (%)**	**Absence (%)**	**Presence (%)**	**Absence (%)**
Anger	6.80	93.20	10.50	89.50
Confusion	13.70	86.30	21.10	78.90
Depression	2.70	97.30	1.80	98.20
Fatigue	46.60	53.40	52.60	47.40
Vigor	23.30	76.70	17.50	82.50
No changes	12.30	87.70	8.80	91.20

*n = 130*.

The comparison between the groups for presence or absence of one specific category of mood state (i.e., anger, confusion, depression, vigor, and no changes) shows no differences for any category when the relative weight was compared. However, when the intensity of RWL strategies was analyzed, a significant difference was found for the intensity of use of training with plastic or thick clothes in the mood state category of fatigue (*U* = 1080.00, Z = −1.97, *p* = 0.04) where the group reporting fatigue showed higher intensity in this practice.

Additionally, differences between groups in the amount of RWL strategies used by the athletes were observed for the categories of vigor and fatigue, where the group reporting the presence of fatigue used more RWL strategies in comparison with the group not-reporting that mood state category. On the other hand, the group reporting the presence of vigor showed lower amount of RWL strategies in comparison with the group not reporting this category of mood state (results are shown in Table 10).

**Table 10:** Comparison between groups of the relative weight loss of athletes of wrestling and taekwondo divided by the presence or absence of mood states as consequence of RWL strategies.

**Mood states**	**Presence, *mean rank***	**Absence, *mean rank***	***U Mann-Whitney***	***Z***	***p***
	**Amount of RWL strategies used**				
Anger	67.27	68.08	674.00	−0.06	0.95
Confusion	62.39	69.15	1159.00	−0.76	0.45
Depression	53.17	68.34	153.50	−0.66	0.50
Fatigue	80.57	55.98	1447.50	−3.68	**0.00**
Vigor	52.83	71.797	1048.50	−2.27	**0.02**
No changes	54.00	69.88	728.00	−1.54	0.12

*RWL, Rapid Weight Loss. Bold values indicate significant p < 0.05*.

Tables 11, 12 report the presence or absence of mood state in the groups of athletes of wrestling and tkd, where fatigue was the greater reported mood in both disciplines, in men and women groups. No differences in the proportion of presence/absence of the mood states were found between sport disciplines nor between females and males.

**Table 11:** Comparison between disciplines in the presence or absence of mood states as consequence of RWL strategies.

**Mood states**	**Wrestling**		**Tkd**		**X^2^ of Pearson**	***p***
	**Presence, %**	**Absence, %**	**Presence, %**	**Absence, %**		
Anger	2.22	28.14	5.92	63.70	0.54	0.96
Confusion	2.96	27.40	14.07	55.55	2.20	0.32
Depression	0.74	29.62	1.48	68.14	0.01	0.98
Fatigue	19.11	11.02	30.14	39.70	4.97	0.09
Vigor	3.70	26.66	16.29	53.33	2.24	0.32
No changes	2.22	28.14	9.62	60.00	1.15	0.55

**Table 12:** Comparison between sexes in the presence or absence of mood states as consequence of RWL strategies.

**Mood states**	**Men**		**Women**		**X^2^ of Pearson**	***p***
	**Presence, %**	**Absence, %**	**Presence, %**	**Absence, %**		
Anger	2.96	56.29	5.18	35.55	2.60	0.10
Confusion	8.88	50.37	8.14	32.59	0.57	0.44
Depression	0.74	58.51	1.48	39.25	0.85	0.35
Fatigue	27.94	31.61	21.32	19.11	0.54	0.45
Vigor	12.59	46.66	7.40	33.33	0.19	0.66
No changes	7.40	51.85	4.44	36.29	0.07	0.77

### Individuals Influencing RWL Practice

In this subsection, the results of the analysis regarding the influence of others in the adoption and use of RWL strategies are shown. Percentage of athletes indicating the different levels of influence for all the individuals included as items in the questionnaire are shown in Table 13. Notice that in general the most influential persons are the coaches, the parents and the nutritionists.

**Table 13:** Percentage of participants reporting the different levels of influence of others to adopt and use rapid weight loss strategies.

**People who influence RWL use**	**No influence (%)**	**Slight influence (%)**	**Unsure (%)**	**Some influence (%)**	**Very influential (%)**
Coach	14.00	13.30	6.70	26.00	40.00
Parents	25.30	17.80	11.60	19.20	26.00
Nutritionist	41.20	14.90	7.40	20.30	16.20
Partner	45.90	24.30	5.40	12.80	11.50
Physical trainer	53.40	13.50	11.50	12.80	8.80
Physician	72.50	16.10	3.40	4.70	3.40
Physical therapist	75.90	9.00	8.30	4.80	2.10

The correlation analysis performed for the whole sample to evaluate the association between the level of influence of others to adopt and use RWL strategies and the relative weight lost, revealed a significant positive correlation with the nutritionist (*r*_*s*_ = 0.17, *p* < 0.05), i.e., the more weight lost, the greater the influence of the nutritionist.

Table 14 shows the results of the comparisons between groups for the intensity of the influence of others in the adoption of RWL strategies. Comparisons consisted of the differences between athletes who lost more or <5% of BM. No differences between groups were found, however a marginal difference (*p* = 0.051) was found for the nutritionist likely suggesting a higher influence in the group losing more than 5% of BM.

**Table 14:** Comparison between subgroups (More vs. Less 5% of Body Mass Loss) and the intensity of influence from others to practice RWL strategies.

**People who influence**	**Athletes who lost ± 5% of body mass**				
	**More than 5%, *mean rank***	**Less than 5%, *mean rank***	***U Mann- Whitney***	***Z***	***p***
Coach	72.31	73.50	2520.00	−0.18	0.86
Parents	76.68	66.91	2084.00	−1.43	0.15
Nutritionist	79.39	66.35	2053.00	−1.96	0.05
Partner	73.63	70.83	2392.50	−0.42	0.67
Physical trainer	77.18	69.15	2239.00	−1.24	0.21
Doctor	73.88	71.51	2437.00	−0.42	0.67
Physical therapist	74.05	67.84	217.00	−1.19	0.23

*RWL, Rapid Weight Loss*.

Table 15 shows the comparison between disciplines in the intensity of influence from others to adopt the practice of RWL. We found that partner influence was greater in the discipline of wrestling than tkd, but coach, parents and physical therapist were more influential in tkd respect to wrestling.

**Table 15:** Comparison between wrestling and taekwondo in the intensity of influence from others to practice RWL strategies.

**People who influence**	**Wrestling, *mean rank***	**Taekwondo, *mean rank***	***U Mann-Whitney***	***Z***	***p***	***Hedges' g***
Partner	88.73	68.08	1691.50	−2.88	**0.004**	0.42
Coach	63.91	80.63	1859.00	−2.27	**0.023**	0.42
Parents	48.00	84.50	1122.00	−4.90	**0.000**	0.93
Physical therapist	63.31	77.35	1814.00	−2.48	**0.013**	0.40

*Bold values indicate significant p < 0.05*.

No differences between sexes in the influence from others to adopt this practice was found.

### Performance Perception as Consequence of RWL

Athletes were asked to indicate whether RWL practices influence their sport performance. Responses were categorized into negative influence or no influence. Among the athletes using RWL strategies (*n* = 130), 76.10% perceived a decline in their performance, while the 23.80% did not perceive changes.

Of 130 athletes, in the group that loses <5% of BM, 68.00% perceive a decline in their sports performance, while 31.90% mention that it does not affect them. On the other hand, of the group of athletes who lose more than 5% of their BM, 86.20% perceive that it affects their performance and 13.80% mentions that it does not have an influence.

In other hand, of 135 athletes in both disciplines, 8.88% of wrestling did not perceive a decline in their sport performance while 25.18% did. In tkd, 50.37% perceived a decline in their sport performance while 15.55% did not.

Finally, 44.40% of men and 31.10% of women perceived a decline, while 0.14% of men and 10.37% of women did not perceive changes.

### Association Between Sport Experience, Success, and RWL

The aim of these analyses was to evaluate the association between the sport experience, success and RWL, to contribute to the clarification of previous contradictory findings in this regard. A negative correlation was found (*r*_*s*_ = −0.18, *p* < 0.05) between sport experience index and the relative weight lost, i.e., the more experience the athletes have, the less weight they lose before a competition.

Regarding the association between the sport experience index and the intensity in the use of certain RWL strategies, our results showed a negative correlation between the sport experience index and intensity of use of saunas (*r*_*s*_ = −0.39, *p* < 0.01), training intentionally in hot rooms (*r*_*s*_ = −0.37, *p* < 0.01) and use of plastic or thick clothes even without training (*r*_*s*_ = −0.51, *p* < 0.01), all of them dehydration strategies.

The success index did not show significant correlations with the quantity of strategies, relative weight lost, nor the frequency of the use of strategies.

## Discussion

The aim of this study was to implement a multilevel evaluation of RWL practice, by means of a questionnaire, in order to understand from a multilevel point of view the factors involved in this practice and how they are associated.

Currently, strategies for RWL represent a typical and well-established procedure prior to competition in most combat sports, even knowing the harmful health risks and consequences that can even lead to death (Franchini et al., 2012).

Reports in different combat sports (judo, tkd, karate) have found a high prevalence of use of RWL (66–94%) in Israeli, Iranian and Malaysian athletes (Kordi et al., 2011; Berkovich et al., 2016; Cheah et al., 2019). In line with previous findings, the results of our study show a high prevalence of about 96% of use of RWL strategies in the sample of Mexican athletes.

Previous literature pointed to a higher incidence of negative effects when athletes lose more than 5% of their BM (Artioli et al., 2010b,c; Franchini et al., 2012); however the incidence of losing more than 5% of BM across these populations has not been reported. One of the novelties of our study is that we differentiate between athletes losing more or <5% of BM by calculating the relative weight lost. Our results showed a prevalence of 57.4% of athletes losing more than 5% of their BM. This finding highlights the urgent need to implement actions to prevent the use of RWL strategies and their negative outcomes.

On the other hand, authors such as Artioli et al. (2010b), mention that the athletes of wrestling having a smaller number of weight categories can lead athletes to use strategies for RWL since there is a greater weight interval between them. Other authors (Kiningham and Gorenflo, 2001; Oppliger et al., 2003; Alderman et al., 2004; Viveiros et al., 2015) confirm the prevalence of 40 to 90% of use of RWL among high school, collegiate and international wrestlers.

The prevalence of use of RWL strategies in different combat sports (judo, tkd, karate, wrestling and boxing) ranges from 70 to 80% (Kordi et al., 2011; Brito et al., 2012; Berkovich et al., 2016), specifically in the study of Reale et al. (2018) were the most prevalent wrestling and tkd athletes, our results are similar to this study since wrestlers presented a prevalence of 98% while tkd athletes presented 94.60%.

In our study the mean age onset of RWL use was 9.9 ± 5.9 years with a range ranging from 7 to 17 years. This result is consistent with those reported in previous literature: in Brazilian athletes the mean age was 11.0 ± 2.5 with a range of 6 to 24 years old (Brito et al., 2012); in the study of Kordi et al. (2011), they found a mean age of 14.2 ± 2.8 years (range 5 to 29 years); however the practice of RWL in athletes younger than 7 years old belonging to the National Collegiate Athletic Association has been documented (Oppliger et al., 2003).

It is documented that the practices of RWL strategies at an early age have a negative impact on the growth and development of the athlete, hormonal imbalance, increased risk of injury due to bone reduction and may also present a higher risk of weight management-related problems throughout their lives (Roemmich and Sinning, 1997; Kowatari et al., 2001; Prouteau et al., 2006; Green et al., 2007; Artioli et al., 2010b; Reljic et al., 2016; Nascimento-Carvalho et al., 2018). Therefore, it is important to start with actions at an early age for the development of athletes taking care of their health and sport performance.

Also, it is known that a small reduction in the amount of body fat in 7 days by means of energy restriction through partial or total reduction of food and fluids results in significant reductions in lean mass and body fluid (Jlid et al., 2013). In our study, the days prior to competitions that the athletes carry out RWL strategies was 9.9 ± 5.9.

While in the study of Smith et al. (2001), boxers reported that prior to 7 days before competition they use active methods such as increased exercise, as the competition approaches they are replaced by passive methods such as restriction of food and fluids, losing ~5.20 ± 0.40% of their BM. This is similar to our results where the percentage of relative weight lost was 5.21 ± 3.99.

In terms of the preferred RWL strategies by the athletes, our results are similar to those previously reported, for instance the results of Aghaei et al. (2011) and Oppliger et al. (2003) with Iranian and American athletes, respectively. They found that they commonly practice different methods for severe dehydration by the use of wet or dry saunas, training in heated rooms, and training in plastic or thick clothes, in addition to restricting fluid intake in the last hours before weighing. In the study of Oppliger et al. (2003) the primary methods of weight loss reported were gradual dieting (79.4%) and increased exercise (75.2%). However, 54.8% fasted, 27.6% used saunas, and 26.7% used rubber/plastic suits at least once a month. In our study, the main strategies that athletes used were dehydration and food restriction, such as increasing exercise, training with plastic or thick clothes, fluid restriction and omitting food. These results are also similar to Reljic et al. (2016) and Smith et al. (2001).

In addition, strategies like skipping meals, fasting, fluid restriction and spit out were the most used in Brazilian athletes of tkd who lost between 3 and 7% of their BM, these results are similar to ours (Silva Santos et al., 2016).

Common RWL methods used by Malaysian athletes were exercising more (69.8%), gradual dieting (51.1%), and training in heated rooms (20.8%) (Cheah et al., 2019), while food and fluid restriction in combination with increased energy expenditure were the preferred methods of weight loss employed by tkd British athletes (Fleming and Costarelli, 2009).

However, dividing the sample into subgroups (more/<5% of their BM) allowed us to identify that those athletes who are in danger because they lose more than 5% of their BM tend to use certain strategies more frequently (increased excerise, training with plastic clothes, fluid restriction, spit out, and use plastic clothes even without training) than those who lose less.

These findings allow us to have a clearer view of the strategies used and to evaluate the level of harm impacting their health and thus be able to establish a probable plan that can directly tackle and prevent negative consequences that RWL encompasses.

Previous studies list the strategies used by athletes, but just a few of them make a distinction in the number of strategies used by the athletes on the basis of the weight they are losing, i.e., whether they are losing more or <5% of BM. In our results, we found that those athletes losing more weight were frequently using several RWL strategies; therefore, we could expect a greater negative effect at muscular level, greater loss of fluids, being more likely to suffer an acute health episode. It is also known that the use of RWL strategies has a negative impact on growth and development (Roemmich and Sinning, 1997), as well as problems related to life-long weight management (Saarni et al., 2006; Artioli et al., 2010b).

Actually, the consequences of dehydration have been reported at a physical level in several studies such as Zubac et al. (2017), where they found that European boxer athletes reported muscle fatigue and/or symptoms of weakness, and onset of muscle pain caused by RWL strategies. These types of ailments could affect sport performance and especially expose athletes to injuries (Kowatari et al., 2001; Smith et al., 2001; Prouteau et al., 2006; Green et al., 2007) during competitions and even in training.

In our study the symptoms that correlated with the highest amount of weight loss were the general condition, breathing and blood pressure, reporting symptoms such as thirst, irritability, weakness, rapid breathing, dizziness, blurred vision, and nausea. It seems that our evaluation was sensitive enough to distinguish the main physiological effects of RWL as a function of the amount of weight lost in our sample.

In addition, in our study the athletes reported a negative change in their mood state with the increase of RWL. Regarding the evaluation of mood state changes as a consequence of RWL practices in the combat athletes, we found that the group who reported vigor shows less use of RWL strategies in comparison to the group not reporting vigor. On the other hand, those participants reporting fatigue-tiredness displayed higher use of RWL strategies than participants who did not report this mood state.

On the other hand, there is evidence of differences between men and women, the latter may be influenced by anxiety generates by the existence of the weight divisions themselves, including the weigh-in process. In contrast, in men, the total emotional disturbance is affected by the practice of RWL; however, in both sexes, fatigue and tension increased when practicing RWL (Yoshioka et al., 2006). In our study, no differences were found between men and women.

In other study, Landers et al. (2001) mentions that the state of positive affection decreased when performing a food restriction hour prior to the competition. On the other hand, Kordi et al. (2011) mentions that there was an increase in fatigue and depression in wrestlers through a self-report. Marttinen et al. (2011) also mentions an increase in confusion on wrestlers. On the contrary, Seyhan (2018) mentions that there is no positive or negative effect in the mood state of vigor, anger, tiredness or stress detected through an *ad hoc* questionnaire. In our study, tkd athletes' reports more mood state of fatigue, vigor and anger, but confusion was more reported by wrestlers.

Our results indicated feeling weak, tired and with muscular pain in the lower train in athletes when using RWL strategies, and are also supported by the findings of Cheah et al. (2019) where athletes reported to perceive adverse effects of RWL on mood state; the most frequent responses included fatigue (69.2%), decreased vigor (50.8%), and muscle cramps (46.2%). Additionally, Kordi et al. (2011) found that the majority of the wrestlers who quickly lose weight reported weakness, fatigue, and myalgia.

Contrary to our results and the results of Kordi et al. (2011) and Cheah et al. (2019), Yang et al. (2018), who investigated the impact of RWL (5% within 3 days) on athletic performance associated hemorheological properties considering relevant recovery time (16 h and between simulated matches). However, it is important to mention that the sample in this study was very small involving five male athletes.

There are three positions on sport performance and RWL. The first posits an improvement in sport performance (Reale et al., 2016b; Coswig et al., 2018), the second suggests no changes (Choma et al., 1995; Yang et al., 2018), while the third supports a decrease in sport performance (e.g., Camarço et al., 2016; de Fortes et al., 2017; Zubac et al., 2017).

Our results support the last; no relationship was found between the relative weight lost quickly and the competitive success, because no correlation was found among these variables, similar to results reported by Zubac et al. (2017). However, Reale et al. (2016b) mentions that RWL is positively related to competitive success measured by the number of medals won. As a future proposal, a longitudinal study is recommended to verify this data.

An important aspect of a comprehensive point of view of the RWL phenomenon is the social influence in the adoption of this practice. Kordi et al. (2011) reported that athletes used to receive information on weight reduction from different sources, and the most important were the coach (57%), other athletes (28%), parents (6%), doctor (3%), nutritionist (2%), and other people such as friends and athletes in other teams (4%). In our study, we found, as in the Kordi et al.' study, that the most influential person was the coach (86%), which makes sense considering the leader role of the coach in the context of the sport team. Alternatively Cheah et al. (2019) mentioned that the physical trainers and training colleagues are commonly rated as the people having the most influence on the use of RWL by the athletes. Another example is the study by Berkovich et al. (2019) in which they examined coaches' attitudes, perceptions and practices regarding RWL strategies, and they found that more than 90% of the participants reported that they usually supervise athletes through the weight loss process and recommend the gradual diet used in combination with dehydration.

Impressively, following the influence of the coach, our results showed that parents (74.7%), and nutritionists (58.8%) are the second and third most influential persons to adopt RWL strategies, respectively. Our results regarding the role of the parents on RWL adoption may be due to a combination of the athletes' age (7–24 years) which cover the child and youth categories and the context-related implicit and explicit cultural rules where parents play a fundamental role in the young athlete's behavior. These context-dependent differences influencing RWL should be considered in the design of strategies for the prevention of RWL practices.

In the study of Park et al. (2019), they reported that athletes who used more strategies for RWL mainly received the influence from social networks, while the athletes who used fewer strategies received nutritional advice. Our study differs in that the nutritionist was one of the most influential people for the adoption of RWL strategies and the physical trainer was the person who was least influential for the adoption of this practice. Our hypothesis is that they who are most concerned about sports performance and know the negative consequences of practicing such strategies.

Artioli et al. (2010b) reported that the main structural factors driving weight loss behaviors are the weight grading system, programming and organizing patterns for competitions. Therefore, other authors mention that RWL could be eradicated by changing the rules of competitive events (Alderman et al., 2004; Artioli et al., 2010a,c; Pettersson et al., 2013; Khodaee et al., 2015; Brandt et al., 2018; Reale et al., 2018; Berkovich et al., 2019).

Considering how difficult this proposal can be, we are committed to educating athletes from an early age as proposed by Calvo-Rico et al. (2013). Since such practices are rooted in the culture of sport, particularly in combat sports, psycho-educational interventions and programs must be created focused on coaches and by creating a sense of identification among athletes, with the support of social figures and influential sport mates, in the behavior of athletes, considering that the people who have the most influence in the teaching of strategies are usually the coaches and former athletes and because the coach is generally the most frequent source of information for athletes.

Educational programs should focus on providing coaches with explanations of how to adequately advise athletes for healthy weight management (Kiningham and Gorenflo, 2001; Kordi et al., 2011; Pettersson et al., 2013; Dubnov-Raz et al., 2015; Khodaee et al., 2015; Do Nascimento et al., 2020). Interventions of this type should act from a multidisciplinary approach, considering the relevance of the associations of all the factors involved.

The team should involve a nutritionist (which, in our study, showed an important relevance in RWL adoption), physician, physiotherapist, physical trainer, etc., in order to reach a successful outcome. Likewise, parents must be involved, since they have a higher intensity of influence for the adoption of RWL strategies, and it is known the parents also have a higher influence in the development of the athletes, especially at younger ages (Gimeno-Marco, 2003).

The relevance of our study is that this approach allowed us to create a comprehensive view of RWL in the sport of wrestling and tkd in Mexican athletes, at individual and group levels. Among the advantages of our study we could highlight: the identification of subgroups losing more or <5% of their BM; the number of strategies they use, as well as the severity of the use; evaluation of the incidence of the practice, not only the physiological effect, but also at psycho-emotional and the perception of the sport performance, providing information that contributes to the dispute about whether or not RWL influence performance; and finally the measure of the level of influence of others to adopt RWL practices.

Furthermore, this study allowed us to obtain a clear picture about how all these factors are related. The relevance of the latter lies in the design and implementation of socio-educational and psychological interventions and programs aimed to prevent RWL practices and their negative effects. Taking back our study the effectiveness of these programs depend on an adequate and comprehensive diagnosis and understanding of the interaction of these factors (Brown et al., 2011; Holmboe and Durning, 2020). These programs should have as main agents the coach, parents and nutritionists, and not only the athlete, from a multidisciplinary perspective.

Finally, our study may constitute a pioneering study for others, where comprehensive evaluation with objective and longitudinal measurements should be proposed, reducing the risks in the athlete's health and optimizing his/her sport performance through the implementation of psychoeducational programs.

## Limitations

Since in our study weight variables were self-reported, it is important for further studies to obtain an objective measure of the weight variations along the season. Also, the age range in our study confirms the early adoption of RWL strategies, as previously reported in the literature, however it is important, when social influence or mood state are evaluated, to consider the influence of age and psychological maturity on these dimensions; further studies could analyze this phenomenon by age groups. Finally, just having two sports makes it necessary to compare with other disciplines.

## Data Availability Statement

The raw data supporting the conclusions of this article will be made available by the authors, without undue reservation.

## Ethics Statement

The studies involving human participants were reviewed and approved by Ethical Committee of Centro de Investigación Transdisciplinar en Psicología of the Universidad Autónoma del Estado de Morelos. Written informed consent to participate in this study was provided by the participants' legal guardian/next of kin.

## Author Contributions

CC-P, JS-L, and JL-W contributed to the conception and design of the study, organized the database, performed the statistical analysis, and wrote sections of the manuscript. CC-P contributed to the data collection and wrote the first draft of the manuscript. All authors contributed to the manuscript revision and read and approved the submitted version.

## Conflict of Interest

The authors declare that the research was conducted in the absence of any commercial or financial relationships that could be construed as a potential conflict of interest.
