# Supplementary material for: Multilevel Evaluation of Rapid Weight Loss in Wrestling and Taekwondo
Source: Front Sociol. 2021 Apr 9;6:637671. doi: 10.3389/fsoc.2021.637671 (PMC8062768; doi:10.3389/fsoc.2021.637671)
Supplement: Supplementary file 1 [file Table_1.docx]

**SUPPLEMENTARY MATERIAL**


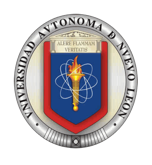

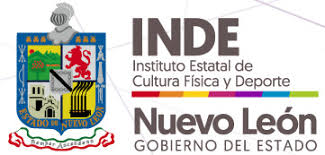

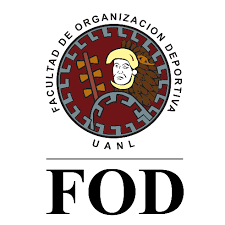


DATOS GENERALES

Fecha: __/__/__

1. Iniciales de nombre completo:_________________________________________________
2. Edad: _____Género: ____F____M
3. Peso: _______kg Estatura: ________cm (Actualmente).
4. Deportes que practicas: ____________________________________________________

**Nota.** A partir de este momento, te pedimos que tus respuestas se enfoquen al deporte por el cual estás siendo evaluado.

1. En qué división de peso compites: ___________
2. A qué edad comenzaste a **practicar**: _________
3. A qué edad comenzaste a **competir** en el deporte que practicas: _______
4. Por favor, indica cada uno de los logros y participación en el deporte que practicas:

Estatal: ( ) Participación sin medalla ( ) Gané medalla ( ) Nunca he participado

Regional: ( ) Participación sin medalla ( ) Gané medalla ( ) Nunca he participado

Nacional: ( ) Participación sin medalla ( ) Gané medalla ( ) Nunca he participado

Internacional: ( ) Participación sin medalla ( ) Gané medalla ( )Nunca he participado

1. En el último año, cuántas veces has competido en el deporte que practicas (incluyendo competencias no oficiales): ____
2. En cuántas competencias has ganado medalla en el último año (incluyendo competencias no oficiales): ____
3. ¿Has cambiado de división (peso) en los últimos dos años?

( ) Si, en cuáles categorías competiste?________________

( ) No, he competido en los últimos dos años en la misma categoría (pasa a la pregunta 13).

1. Menciona cuál o cuáles fueron los motivos por los que cambiaste de categoría: ____________________________________________________________
2. ¿Cuál es tu peso cuando estas fuera de temporada? ______kg.
3. Cuál es la mayor cantidad de peso en kilogramos que has perdido de forma rápida para una competencia en tu carrera deportiva: ____kg
4. Usualmente, ¿En cuántos días realizas la pérdida rápida de peso cuando se acerca una competencia? ____días
5. Usualmente, cuánto peso (kg) es el que recuperas en la semana siguiente de la competición: ____kg/semana.
6. En las tablas de abajo se muestran algunas **estrategias** para la pérdida de peso.

Marca con una X qué tan frecuente las has utilizado.

|  | Nunca la he utilizado | Ya no la utilizo | Casi nunca | Algunas veces | Siempre |
| --- | --- | --- | --- | --- | --- |
| Dieta gradual (pérdida de peso en 2 o más semanas) |  |  |  |  |  |

Marca con una X la frecuencia de utilización de las siguientes estrategias para la pérdida rápida de peso, al menos 5 días antes de la competencia.

|  | Nunca la he utilizado | Ya no la utilizo | Casi nunca | Algunas veces | Siempre |
| --- | --- | --- | --- | --- | --- |
| Omitir 1 o 2 comidas |  |  |  |  |  |
| Ayuno (mínimo 16 horas sin alimento) |  |  |  |  |  |
| Restricción de líquidos |  |  |  |  |  |
| Incremento de ejercicio (practicar más de lo usual) |  |  |  |  |  |
| Entrenar intencionalmente en cuartos calientes |  |  |  |  |  |
| Saunas |  |  |  |  |  |
| Entrenar con plásticos o ropa gruesa |  |  |  |  |  |
| Usar plásticos o ropa gruesa durante todo el día (incluso cuando no hago ejercicio) |  |  |  |  |  |
| Escupir |  |  |  |  |  |
| Laxantes |  |  |  |  |  |
| Diuréticos |  |  |  |  |  |
| Pastillas para adelgazar |  |  |  |  |  |
| Vomitar |  |  |  |  |  |

1. Usualmente, ¿Cuántos kilos pierdes antes de la competencia utilizando estrategias para la perdida rápida de peso? ____kg
2. A qué edad comenzaste a practicar estrategias para la pérdida rápida de peso previo a tu competencia: ____años
3. ¿Quiénes y con qué frecuencia consideras que han influido para utilizar alguna de las estrategias de pérdida rápida de peso? (Coloca el número que creas más conveniente en el paréntesis de cada una de las personas que se mencionan).

1 no influye 2 ligera influencia 3 inseguro 4 algo de influencia 5 muy influyente

( ) compañero ( ) entrenador

( ) preparador físico ( ) padres

( ) médico ( ) nutriólogo

( ) otro:____________________ ( ) fisioterapeuta

1. Describe la forma en la que fluctúa/cambia tu peso desde la etapa general a la etapa competitiva: ________________________________________________________________________________________________________________________________________________
2. Describe cuál es tu sensación al utilizar estrategias para la perdida rápida de peso: ________________________________________________________________________________________________________________________________________________
3. Describe si consideras que el usar las estrategias para pérdida rápida de peso influyó en tu rendimiento: ________________________________________________________________________________________________________________________________________________
4. Fecha de tu próxima competencia_____________________________________________
5. De acuerdo a los siguientes síntomas, marca con una X los síntomas que presentas al practicar estrategias para la perdida rápida de peso en un **periodo pre-competitivo (3-5 días antes de la competencia):**

| Estado general | Sediento, alerta  ( ) | Sediento, alerta, inquieto o letárgico/torpe o irritable al ser tocado  ( ) | Soñolientos, flácidos sudorosos, a veces comatosos/inactividad excesiva, miembros cianóticos/extremidades de cambian de color azul ( ) |
| --- | --- | --- | --- |
| Frecuencia Cardíaca | Normal  ( ) | Rápido y débil  ( ) | Rápido e impalpable a veces  ( ) |
| Respiración | Normal  ( ) | Profunda, puede ser rápida  ( ) | Profunda y rápida  ( ) |
| Ojos | Normales  ( ) | Hundidos  ( ) | Muy hundidos  ( ) |
| Lágrimas | Existen  ( ) | Disminuyen o faltan  ( ) | Faltan  ( ) |
| Mucosas | Húmedas  ( ) | Secas  ( ) | Muy secas  ( ) |
| Orina | Normal  ( ) | Escasa y oscura  ( ) | Disminución intensa o falta de orina  ( ) |
| Presión arterial | Normal  ( ) | Mareo, visión borrosa, náuseas  ( ) | Mareo, somnolencia, debilidad, náuseas, confusión, visión borrosa  ( ) |
